# Evaluation of ERG and SPINK1 by Immunohistochemical Staining and Clinicopathological Outcomes in a Multi-Institutional Radical Prostatectomy Cohort of 1067 Patients

**DOI:** 10.1371/journal.pone.0132343

**Published:** 2015-07-14

**Authors:** James D. Brooks, Wei Wei, Sarah Hawley, Heidi Auman, Lisa Newcomb, Hilary Boyer, Ladan Fazli, Jeff Simko, Antonio Hurtado-Coll, Dean A. Troyer, Peter R. Carroll, Martin Gleave, Raymond Lance, Daniel W. Lin, Peter S. Nelson, Ian M. Thompson, Lawrence D. True, Ziding Feng, Jesse K. McKenney

**Affiliations:** 1 Department of Urology, Stanford University, Stanford, California, United States of America; 2 The Department of Biostatistics, The University of Texas MD Anderson Cancer Center, Houston, Texas, United States of America; 3 Canary Foundation, Canary Center at Stanford, Palo Alto, California, United States of America; 4 Department of Urology, University of Washington Medical Center, Seattle, Washington, United States of America; 5 The Prostate Center at Vancouver General Hospital, University of British Columbia, Vancouver, Canada; 6 Department of Pathology, University of California San Francisco, San Francisco, California, United States of America; 7 Department of Pathology, University of Texas Health Science Center at San Antonio, San Antonio, Texas, United States of America; 8 Eastern Virginia Medical School, Pathology, Microbiology and Molecular Biology, Norfolk, Virginia, United States of America; 9 Department of Urology, University of California San Francisco, San Francisco, California, United States of America; 10 Department of Urology, Eastern Virginia Medical School, Norfolk, Virginia, United States of America; 11 Division of Human Biology, Fred Hutchinson Cancer Research Center, Seattle, Washington, United States of America; 12 Department of Urology, University of Texas Health Science Center at San Antonio, San Antonio, Texas, United States of America; 13 Department of Pathology, University of Washington Medical Center, Seattle, Washington, United States of America; 14 Department of Pathology, Cleveland Clinic, Cleveland, Ohio, United States of America; Innsbruck Medical University, AUSTRIA

## Abstract

Distinguishing between patients with early stage, screen detected prostate cancer who must be treated from those that can be safely watched has become a major issue in prostate cancer care. Identification of molecular subtypes of prostate cancer has opened the opportunity for testing whether biomarkers that characterize these subtypes can be used as biomarkers of prognosis. Two established molecular subtypes are identified by high expression of the ERG oncoprotein, due to structural DNA alterations that encode for fusion transcripts in approximately ½ of prostate cancers, and over-expression of SPINK1, which is purportedly found only in ERG-negative tumors. We used a multi-institutional prostate cancer tissue microarray constructed from radical prostatectomy samples with associated detailed clinical data and with rigorous selection of recurrent and non-recurrent cases to test the prognostic value of immunohistochemistry staining results for the ERG and SPINK1 proteins. In univariate analysis, ERG positive cases (419/1067; 39%) were associated with lower patient age, pre-operative serum PSA levels, lower Gleason scores (≤3+4=7) and improved recurrence free survival (RFS). On multivariate analysis, ERG status was not correlated with RFS, disease specific survival (DSS) or overall survival (OS). High-level SPINK1 protein expression (33/1067 cases; 3%) was associated with improved RFS on univariate and multivariate Cox regression analysis. Over-expression of either protein was not associated with clinical outcome. While expression of ERG and SPINK1 proteins was inversely correlated, it was not mutually exclusive since 3 (0.28%) cases showed high expression of both. While ERG and SPINK1 appear to identify discrete molecular subtypes of prostate cancer, only high expression of SPINK1 was associated with improved clinical outcome. However, by themselves, neither ERG nor SPINK1 appear to be useful biomarkers for prognostication of early stage prostate cancer.

## Introduction

Based on high incident rates of 230,000 cases per year, significant mortality rates of 29,000 men yearly, and a relatively slow natural history, prostate cancer should be an ideal target for screening interventions to impact survival [1]. The drop in death rates from 40,000 cases per year to current rates suggests that PSA screening has made an impact on prostate cancer mortality [2]. However, results from prospective randomized screening and surgical intervention trials, particularly the Prostate Lung, Colon and Ovarian (PLCO) and PIVOT trials in North America, have raised questions as to the effectiveness of screening to decrease deaths [3, 4]. While the ERSPC trials and SPCG-4 conducted in less heavily screened populations of Europe showed benefits to PSA screening and surgical treatment for prostate cancer specific mortality [5, 6], taken together all of the trials highlight potential over-screening and over-treatment of prostate cancer as major risks, particularly in light of the morbidities associated with prostate cancer treatments [7].

Much as therapies targeted to discrete molecular lesions are making an impact in the management of advanced cancers, the concept of using molecular markers to identify aggressive and potentially lethal cancers has gained traction in managing early stage prostate cancer [8]. Evidence from the intervention trials as well as observations of the high prevalence of prostate cancer at autopsy suggest that there is a very large pool of prostate cancers that should not be diagnosed and do not require therapy [9]. Current clinical markers, including tumor stage, serum PSA levels and biopsy Gleason score, lack sufficient predictive power across all clinical scenarios to confidently select patients who do not harbor future risk of disease progression and can be safely observed; therefore, identification of molecular features that correlate with aggressive disease is a high priority.

To address the need for validation of candidate biomarkers of disease aggressiveness, we have developed a prostate cancer tissue microarray (Canary prostate TMA). The TMA resource was constructed at 6 participating centers using a common protocol of radical prostatectomy specimens with complete clinical data and long-term follow-up [10]. These TMAs had a rigorous statistical design including random case selection, case sampling schemes to minimize spectrum biases, and oversampling of cases in specific groups of interest to help in identifying biomarkers that best predict failure after radical prostatectomy, a surrogate for aggressive disease.

Prostate cancers are characterized by over-expression of the ETS transcription factor ERG as a result of a somatically acquired fusion event to the regulatory region of the TMPRSS2 gene [11]. These gene fusions are found in nearly half of prostate cancers and are thought to constitute a distinct molecular subtype of the disease. Over-expression of SPINK1 has been described in cancers lacking the TMPRSS2-ERG fusion and has been reported to identify a subset (approximately 5–10%) of prostate cancers that behave more aggressively [12]. Conflicting results have been reported on whether ERG and SPINK1 over-expression is associated with adverse outcome (summarized in [13] and [14]). We tested whether either biomarker, whether alone or in combination, predicted outcomes after radical prostatectomy in our multi-institutional TMA resource.

## Materials and Methods

### Ethics Statement

Tissue blocks and accompanying clinical data were collected at each of the participating sites (Stanford University, University of California San Francisco, University of Washington, University of British Columbia, University of Texas Health Sciences Center at San Antonio, Eastern Virginia Medical School) under a research protocol developed by the investigators with IRB approval at each institution. The approved protocols included sharing of de-identified data and samples and correlation of clinical data with biomarker data acquired from the TMAs. A materials transfer agreement was developed jointly and approved at each site for sharing of tissue microarrays and tissue samples.

### TMA cases and construction

For case selection, de-identified clinical data were submitted to the statistical core (lead statistician ZF) for random case selection. Constraints were placed on selection such that recurrent cases in patients with Gleason score 3+3 = 6 and non-recurrent cases in those with Gleason score 4+4 = 8 were oversampled. In addition, cases were selected to attempt to balance the number of recurrent and non-recurrent cases at each site. Details of case selection, tissue microarray construction and statistical considerations have been detailed elsewhere [10].

Once cases were selected, tissue blocks were obtained at each site. In cases where tissue blocks were not available, additional cases were selected in accord with a random list generated by the data repository. Tissue microarrays were constructed at each participating site in accord with a standard protocol. Briefly, 3 cores of the highest grade cancer from the largest cancer area were harvested as 1 mm cores and transferred to the recipient block. In addition, one core of histologically normal prostate tissue was included from each case. Once constructed, the TMAs were baked and stored under nitrogen gas at each site.

### Immunohistochemistry (IHC)

Freshly cut 5 micron sections from each site were shipped to Stanford University for immunohistochemical staining. ERG immunohistochemistry was performed using a commercial rabbit monoclonal antibody to ERG (clone EPR3864; 1:100; Epitomics, Burlingame, CA, USA) as described previously [15]. SPINK1 expression was assessed with a mouse monoclonal antibody (1:50 dilution; H00006690-M01, Abnova) [14]. In addition, TMAs were stained with hematoxylin and eosin (H & E) as well as immunohistochemical staining using a mouse monoclonal antibody (34bE12, Dako) for high molecular weight keratins (HMWK). The H&E and HMWK slides were scanned to digital images using a Leica SL801 autoloader and SCN400 scanning system (Leica Microsystems; Concord, Ontario, Canada) at magnification equivalent to ×20 and images of individual cores were viewed and scored using the SlidePath digital imaging hub (DIH; Leica Microsystems) of the Vancouver Prostate Centre and share online with Canary pathology team. Scoring was performed on-line for the presence of cancer in each core on the TMA, and only cases with cancer were scored for ERG and SPINK1 (all performed by a single pathologist: JKM).

TMAs from one institution had technically insufficient staining for ERG and were, therefore, excluded from the analysis, leaving a total of 1067 patients who were included in this analysis. For SPINK1, the percentage of neoplastic cells demonstrating cytoplasmic staining were recorded for each individual core based on distinct expression patterns that were recognized: 0- no staining, 1- less than 50% of cells staining in scattered individual cells, 2- less than 50% of cells staining in complete glands, 3–50–80% of cells staining, 4- greater than 80% of cells staining. The SPINK1 staining score 4 was based on identical criteria utilized by Tomlins et al. as an independent predictor of biochemical recurrence [12]. For ERG, the staining was scored for each individual core as follows: 0- no staining, 1- faint nuclear staining visualized at high power magnification, 2- strong nuclear reactivity easily seen at low power magnification (100X magnification or less). The criteria utilized for an ERG score 2 were identical to those that have been shown to correlate with fusion status [15, 16]. For each antibody, the highest score recorded for a case in any of its three individual cores was utilized in the statistical analysis for that individual patient.

### Statistical methods

The primary endpoint of this analysis was post-surgery recurrence-free survival (RFS) where the baseline was set at the date of surgery. RFS was defined as absence of PSA (biochemical) recurrence, local recurrence, prostate cancer metastases, or death from prostate cancer, with events scored at the earliest date noted after surgery. Disease-specific survival (DSS), defined as death from prostate cancer or development of advanced metastatic disease, and overall survival (OS) were secondary endpoints. SPINK1 and ERG score for each patient was the maximum score of all the cores from that patient as defined above.

Summary statistics of patients’ SPINK1, ERG, and combined staining status were provided in frequencies and percentages. Fisher’s exact test was used to assess the association between ERG and SPINK1 status with each other and with patient characteristics. Kaplan-Meier (KM) method was used to estimate survival endpoints by patient group. Cox proportional hazard model was used to estimate effects of ERG and SPINK1 on each survival endpoint. Unweighted and weighted analyses were performed, with the latter accounting for the oversampling of patients with recurrence less than 5 years after surgery. All tests were two-sided and p-values of 0.05 or less were considered statistically significant. Statistical analysis was carried out using SAS version 9 (SAS Institute, Cary, NC). Kaplan Meier plots were generated using Spotfire S+ 8.2 (TIBCO Inc., Palo Alto, CA). The complete dataset of clinical, pathological and staining data can be found in S1 File.

## Results

### Patient population

**Fig 1:**
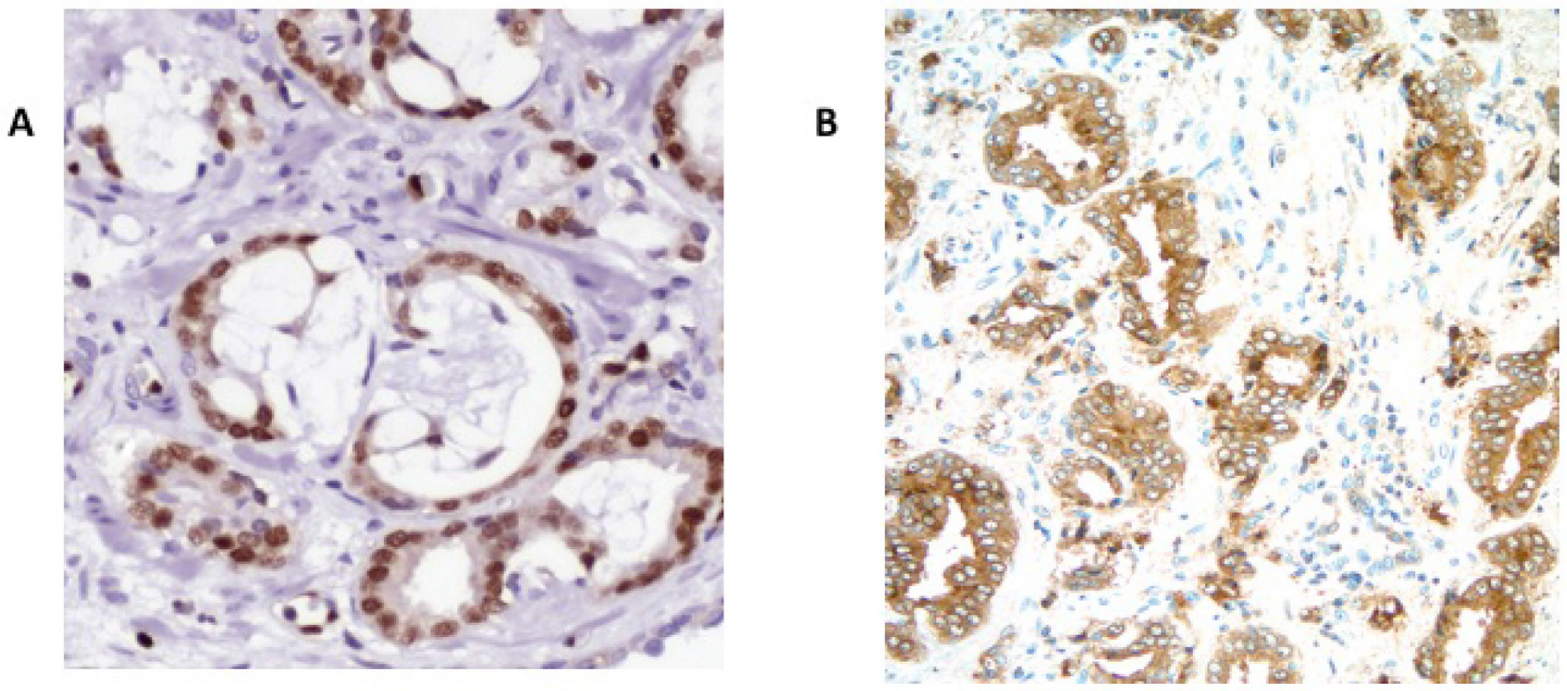
Immunohistochemical staining showing high level expression of A) ERG – nuclear staining, and B) SPINK1 with cytoplasmic staining.

After exclusion of TMAs from 1 study site for technical issues, a total of 1067 patients had evaluable ERG or SPINK1 status by IHC. The mean age of the entire cohort was 61.7 ± 7.2 (range 35 to 80) and mean PSA was 8.7 ± 8.8. For ERG, a total of 113 cases (11%) did not have evaluable staining data either because of core loss or because lack of cancer in the core samples. Of the remaining tumors, 44% (419/954) showed strong ERG expression (score 3), 53% (506/954) showed no expression (score 0), with the remaining showing faint ERG expression (score 1) (29/954 or 3%) (Fig 1A).

For SPINK1, immunostaining results were available on 90% (963/1067) of cases with 104 cases lacking interpretable staining data. SPINK1 expression was strongly positive (score 4) in 3.4% of cases (33/963) and absent from 86% (826/963) with the remaining 104 (11%) cases showing varying degrees of faint staining (Fig 1B). Of 954 patients with evaluable SPINK1 and ERG staining, 3 cases had strong expression of both SPINK1 and ERG protein, although this overlap was lower than expected by chance (P<0.0001, Fisher’s exact test). Staining results and clinical data are summarized in Table 1.

**Table 1:** Summary of clinical, pathological and staining characteristics.

Variable	Status	Number	Percent
**Gleason Score**	Missing	10	0.94
	≤6	429	40.21
	3+4	387	36.27
	4+3	133	12.46
	10-Aug	108	10.12
**Extracapsular extension**	Missing	9	0.84
	Negative	793	74.32
	Positive	265	24.84
**Surgical margins**	Missing	179	16.78
	Positive	306	28.68
	Negative	582	54.55
**Seminal vesicle invasion**	Missing	14	1.31
	No	984	92.22
	Yes	69	6.47
**ERG staining**	Missing	113	10.59
	0	506	47.42
	1	29	2.72
	2	419	39.27
**SPINK1 staining**	Missing	104	9.75
	0	826	77.41
	1	68	6.37
	2	24	2.25
	3	12	1.12
	4	33	3.09
**Recurrence Free Survival**	No Event	588	55.11
	Event	479	44.89
**Disease Specific Survival**	No Event	1013	94.94
	Mets or Ca Death	54	5.06
**Overall Survival**	Alive	996	93.35
	Dead	71	6.65

### ERG /SPINK1 expression and clinicopathological variables

High-level expression of ERG (score 2) and SPINK1 (score 4) by IHC were tested for their association with clinical and pathologic features (Table 2). Neither ERG nor SPINK1 expression was associated with pathological findings of seminal vesicle invasion, positive surgical margins or extracapsular extension. ERG positive cases were more likely to be lower grade (Gleason score ≤3+4 = 7; P = 0.01, Fisher’s exact test), slightly younger (mean age 60.5 vs. 62.5; P<0.0001, Wilcoxon rank sum test) and have lower pre-operative serum PSA levels (7.9 vs. 9.3ng/ml; P = 0.0003, Wilcoxon rank sum test) compared to ERG negative cases. There were no differences in Gleason score distribution, age or pre-operative PSA levels in the SPINK1 positive and negative cases. When cases were grouped for positive staining for either marker vs. no staining for either marker, positive staining results were correlated with lower Gleason score (Gleason score ≤3+4 = 7; P = 0.03, Fisher’s exact test), age (mean age 60.6 vs. 62.5; P = 0.0001, Wilcoxon rank sum test) and pre-operative serum PSA levels (7.9 vs. 9.4 ng/ml; P = 0.0005, Wilcoxon rank sum test) and this association appeared to be largely driven by ERG positive cases. The presence of extracapsular extension was slightly lower in cases in which either marker was positive (41.4%) compared to cases in which both markers were negative (58.6%) (P = 0.05). However, neither marker alone was associated with extracapsular extension.

**Table 2:** Summary of ERG, SPINK1, and ERG/SPINK1 by pathological features.

Feature	Status	ERG Neg	ERG Pos	P-value	SPINK1 Neg	SPINK1 Pos	P-value	Both Neg	Either Pos	P-value
**Surgical Margin**	Positive	168(61.5%)	105(38.5%)	0.08	264(96%)	11(4%)	1.00	158(57.9%)	115(42.1%)	0.13
	Negative	285(55%)	233(45%)		505(96.2%)	20(3.8%)		270(52.1%)	248(47.9%)	
**Stage**	III/IV	142(61.5%)	89(38.5%)	0.26	227(97.4%)	6(2.6%)	0.31	137(59.3%)	94(40.7%)	0.13
	I/II	302(56.9%)	229(43.1%)		514(95.5%)	24(4.5%)		282(53.1%)	249(46.9%)	
**SVinv**	Negative	482(54.9%)	396(45.1%)	0.09	856(96.6%)	30(3.4%)	1.00	458(52.2%)	420(47.8%)	0.11
	Yes	41(66.1%)	21(33.9%)		61(96.8%)	2(3.2%)		39(62.9%)	23(37.1%)	
**ECE**	Negative	385(54.8%)	318(45.2%)	0.18	682(95.9%)	29(4.1%)	0.10	361(51.4%)	342(48.6%)	0.05
	Yes	146(59.8%)	98(40.2%)		241(98.4%)	4(1.6%)		143(58.6%)	101(41.4%)	
**Gleason Score**	< = 6	190(51.8%)	177(48.2%)	0.01	362(97.1%)	11(2.9%)	0.63	183(49.9%)	184(50.1%)	0.03
	3+4	198(55%)	162(45%)		347(96.1%)	14(3.9%)		186(51.7%)	174(48.3%)	
	4+3	83(66.4%)	42(33.6%)		122(97.6%)	3(2.4%)		80(64%)	45(36%)	
	8–10	61(64.2%)	34(35.8%)		92(94.8%)	5(5.2%)		56(58.9%)	39(41.1%)	

P-values by Fisher’s exact test.

### ERG/SPINK1 expression and clinical outcomes

**Fig 2:**
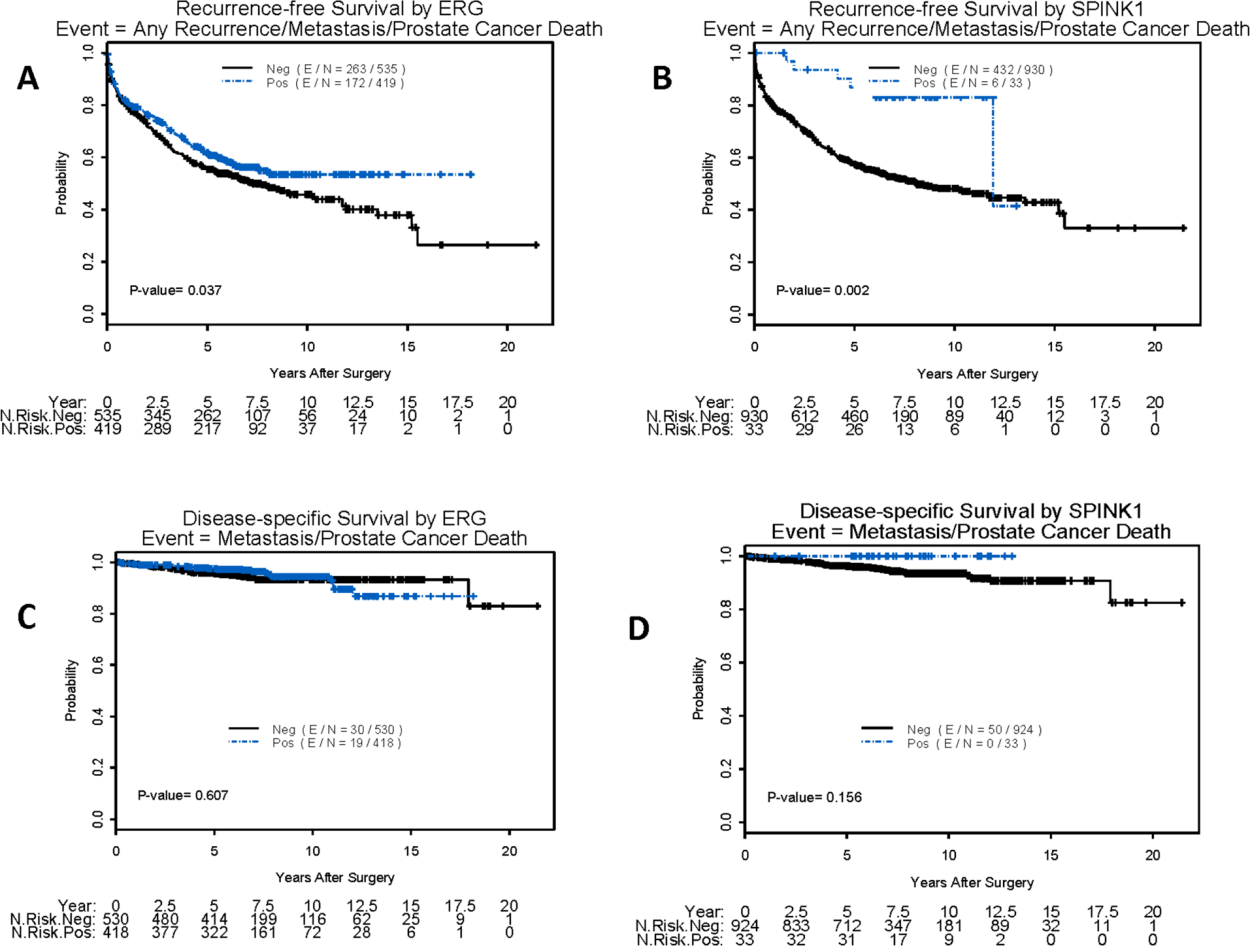
Kaplan-Meier plots of showing the relationship of expression of ERG or SPINK1 and clinical outcome: A) High expression of ERG is associated with improved RFS B) High expression of SPINK1 is associated with improved RFS C) High expression of ERG is not associated with diseases specific survival or development of metastases D) High expression of SPINK1 is not associated with diseases specific survival or development of metastases.

In univariate Cox proportional hazards analysis, positive ERG expression was associated with improved RFS (HR = 1.23; P = 0.04), as was strong positive SPINK1 expression (HR = 3.32; P = 0.004) and positive expression of either marker (HR = 1.33; P = 0.003). However, neither marker, either alone or in combination, was associated with DSS or OS (Table 3). High level expression of ERG (Fig 2A) and SPINK1 (Fig 2B) was associated with improved RFS by Kaplan-Meier analysis, although neither was associated with DSS (Fig 2C and 2D) or OS (not shown).

**Table 3:** Univariate Cox proportional hazard models.

Endpoint	Factor	Comparison	Hazard Ratio	95% LCL	95% UCL	P-value	# Event	# Censored	Total # Patients
**RFS**	ERG	Neg vs. Pos	1.23	1.01	1.49	0.04	435	519	954
	SPINK1	Neg vs. Pos	3.32	1.48	7.42	0.004	438	525	963
	ERG/SPINK1	Neg vs. Pos	1.33	1.1	1.61	0.003	435	519	954
	Margin	Pos vs. Neg	2.03	1.66	2.47	< .0001	395	493	888
	Stage	III/IV vs. I/II	2.4	1.96	2.94	< .0001	385	477	862
	SVinv	No vs. Yes	0.28	0.21	0.38	< .0001	470	583	1053
	ECE	No vs. Yes	0.5	0.41	0.61	< .0001	474	584	1058
	Gleason	3+4 vs. < = 6	1.58	1.27	1.98	0.0001	470	587	1057
		4+3 vs. < = 6	2.7	2.07	3.53	< .0001			
		8–10 vs. < = 6	2.62	1.96	3.52	< .0001			
	Age	1 unit increase	1.01	0.99	1.02	0.43	459	502	961
	Log(pre-op PSA)	1 unit increase	1.96	1.69	2.27	< .0001	431	510	941
**DSS**	ERG	Neg vs. Pos	1.16	0.65	2.07	0.61	49	899	948
	SPINK1	Neg vs. Pos	NA	NA	NA	0.99	50	907	957
	ERG/SPINK1	Neg vs. Pos	1.32	0.74	2.35	0.35	49	899	948
	Margin	Pos vs. Neg	2.44	1.27	4.69	0.0073	37	847	884
	Stage	III/IV vs. I/II	6.7	3.13	14.33	< .0001	35	821	856
	SVinv	No vs. Yes	0.29	0.15	0.57	0.0004	54	994	1048
	ECE	No vs. Yes	0.39	0.22	0.67	0.0007	52	1000	1052
	Gleason	3+4 vs. < = 6	2.55	1.19	5.47	0.02	53	998	1051
		4+3 vs. < = 6	3.56	1.44	8.82	0.006			
		8–10 vs. < = 6	6.88	3.05	15.56	< .0001			
	Age	1 unit increase	1.02	0.98	1.06	0.3	53	902	955
	Log(pre-op PSA)	1 unit increase	2.12	1.49	3.02	< .0001	47	888	935
**OS**	ERG	Neg vs. Pos	0.72	0.41	1.26	0.25	49	893	942
	SPINK1	Neg vs. Pos	0.6	0.19	1.93	0.39	49	901	950
	ERG/SPINK1	Neg vs. Pos	0.63	0.36	1.11	0.11	49	893	942
	Margin	Pos vs. Neg	1.67	0.99	2.83	0.06	56	823	879
	Stage	III/IV vs. I/II	2	1.19	3.38	0.01	57	792	849
	SVinv	No vs. Yes	0.4	0.19	0.85	0.02	57	984	1041
	ECinv	No vs. Yes	0.48	0.28	0.81	0.01	56	989	1045
	Gleason	3+4 vs. < = 6	0.93	0.47	1.84	0.83	58	986	1044
		4+3 vs. < = 6	1.27	0.51	3.17	0.61			
		8–10 vs. < = 6	4.14	2.18	7.89	< .0001			
	Age	1 unit increase	1.07	1.03	1.11	0.0011	58	890	948
	Log(pre-op PSA)	1 unit increase	1.65	1.11	2.44	0.01	38	890	928

LCL = Lower Confidence Limit, UCL = Upper Confidence Limit, RFS = Recurrence Free Survival, DSS = Disease Specific Survival, OS = Overall Survival

To evaluate whether either biomarker provided prognostic information independent of clinical variables, we performed multivariate Cox proportional hazards analysis using a backwards elimination procedure to identify the final model for each endpoint (Table 4). For RFS, absent SPINK1 expression was correlated with worse clinical outcome (HR = 2.84; P = 0.02), as were presence of positive surgical margins, seminal vesicle invasion, higher pre-operative PSA and increasing Gleason score. ERG expression was not associated with RFS, DSS or OS. DSS was associated only with Gleason score and pre-operative PSA and OS were associated only with Gleason score and age. The relatively small number of prostate cancer deaths or metastases (54) and deaths from all causes (71) limited our ability to test the association of the biomarkers with these endpoints. Conclusions from weighted and unweighted analyses were similar with respect to biomarker effects on survival endpoints.

**Table 4:** Multivariate Cox proportional hazard models.

Endpoint	Factor	Comparison	Hazard Ratio	95% LCL	95% UCL	P-value
**RFS** (N = 674,E = 306)	SPINK1	Neg vs. Pos	2.84	1.17	6.90	0.02
	Margin	Pos vs. Neg	1.78	1.41	2.24	<0.0001
	SVinv	Yes vs. No	2.37	1.63	3.43	<0.0001
	Gleason	3+4 vs. < = 6	1.46	1.10	1.95	0.009
		4+3 vs. < = 6	2.09	1.49	2.93	< .0.0001
		8–10 vs. < = 6	1.82	1.26	2.65	0.002
	Log(pre-op PSA)	1 unit increase	1.56	1.31	1.86	< .0.0001
**DSS** (N = 929,E = 46)	Gleason	3+4 vs. < = 6	2.69	1.11	6.49	0.03
		4+3 vs. < = 6	3.67	1.34	10.07	0.01
		8–10 vs. < = 6	6.27	2.41	16.31	0.0002
	Log(pre-op PSA)	1 unit increase	1.80	1.23	2.64	0.003
**OS** (N = 940, E = 58)	Gleason	3+4 vs. < = 6	0.88	0.44	1.73	0.71
		4+3 vs. < = 6	1.11	0.44	2.77	0.82
		8–10 vs. < = 6	3.25	1.70	6.24	0.0004
	Age	1 unit increase	1.06	1.02	1.10	0.006

N = total number of patients, E = number of patients with events

LCL = Lower Confidence Limit, UCL = Upper Confidence Limit

## Discussion

Molecular subtypes of prostate cancer defined by ERG expression do not appear to correlate with clinical outcomes in patients undergoing surgery for localized prostate cancer. On the other hand, we found that high SPINK1 protein expression was associated with lower rates of recurrence after surgery, although SPINK1 overexpression defines only a small subset of prostate cancers (3.4%). ERG and SPINK1 expressing cancers do not appear to be strictly mutually exclusive molecular subtypes, although SPINK1 expression does appear to be uncommon in ERG-expressing cancers. This observation agrees with other studies showing a small subset of tumors expressing high levels of both markers [14, 17].

Studies of the prognostic role of the TMPRSS2:ERG fusion or ERG over-expression have reported associations with worse clinical outcome, improved clinical outcome and a lack of association ([18–26] and summarized in [13] and [27]). In some cases, the discrepant findings can be attributed to small sample sizes or segregation of adverse clinical features in ERG positive tumors or ERG negative tumors by chance. For instance, in our univariate analysis, ERG negative tumors had a slightly worse outcome, but this finding disappeared when we adjusted for age, Gleason score and pre-operative serum PSA levels. While an association between ERG expression and age and serum PSA levels has been observed in previous studies [13, 28] this association is unlikely to reflect prostate cancer biology since the relative frequency of the TMPRSS2:ERG fusions appears to be similar across early stage and metastatic prostate cancer, implying there is no selection of this molecular subtype with progression [29, 30]. It is also possible that the range of associations of the TMPRSS2:ERG fusion or ERG over-expression with prognosis is due to differences in the populations studied or other clinical or pathologic features. For example, ERG fusions and over-expression can vary between different ethnic groups and are less common in transition zone tumors [13, 31, 32]. Prostate cancer outcomes after surgery have been associated with ethnicity and tumor location [33–35]. The size of our cohort and distribution of cases across several institutions, as well as the careful case selection likely minimized these potential biases, and we found no association of ERG expression with clinical outcome. Our data support an emerging consensus that the presence of the TMPRSS2:ERG fusion or ERG over-expression are not associated with more aggressive prostate cancers [13, 27, 36].

High SPINK1 expression was associated with improved RFS in our cohort. This is in contrast with other reports that report high SPINK1 expression associated with worse RFS or null-association [12, 14, 19, 37–39]. It is unclear why SPINK1 expression shows variable results between studies, although it is likely that the small number of SPINK1 positive cases could lead to imbalances in the distribution of clinical risk factors between studies. Given our finding that high expression of SPINK1 is associated with improved outcomes, while others find it associated with worse outcome, our positive association needs to be interpreted with caution.

While ERG status was not prognostic in our cohort, it has been proposed that ERG status might define molecular subtypes that provide context for other biomarkers. For example, PTEN loss has been associated with adverse pathology and worse RFS in ERG overexpressed tumors, but not in ERG negative tumors [17, 23, 40–42]. In addition, increased expression of CRISP3 has been shown to be enriched in high ERG and PTEN expressing tumors and also associated with worse DSS [43]. Low expression of ERG and TERT in urine samples has been associated with improved RFS compared to samples expressing either or both genes [44]. Increased expression of proliferation associated proteins Ki67 and TOP2A has been found to be more highly prognostic in ERG-negative prostate cancers [45]. While loss of expression of p27 has been noted in ERG-negative prostate cancers, p27 loss was not associated with clinical outcomes [46]. Because of the relative infrequency of SPINK1 alterations, it is difficult to assess whether this molecular subclass of tumors can be further subtyped prognostically. ERG and SPINK1 positive tumors have been proposed to describe discrete molecular subtypes of prostate cancer. In our cohort there did not appear to be a significant interaction between these subtype biomarkers. While tumors positive for either of these markers appeared to have improved RFS compared to tumors lacking both, multivariable analysis failed to demonstrate an association between RFS, DSS or OS in marker positive vs. negative cases. Our findings are consistent with a recent publication demonstrating a lack of association with clinical outcome for ERG-positive, ETS-positive, SPINK-positive and marker negative (triple negative) prostate cancers based on gene expression profiling [47]. Much additional work with large clinical datasets, such as ours, will be necessary to test whether molecular subtyping with ERG and SPINK1 will provide clinically or biologically meaningful information in prostate cancer.

While ERG and SPINK1 do not appear to be strong prognosticators, it is possible that they could have other roles as biomarkers, such as in defining molecular subtypes that respond to different therapies (i.e. as predictive biomarkers). For example, in a large cohort (N = 2800) of radical prostatectomy patients, high ERG expression was not correlated with biochemical recurrence, but was correlated with high level expression of the androgen receptor (AR) [36]. This finding suggests that ERG overexpressed tumors might be particularly sensitive to AR inhibition, although this concept has been challenged based on analysis of ERG expression in hormonally treated patients [19]. In addition, TMPRSS2:ERG gene fusions secondary to deletions of chromosome 21q22 and increased copy number of the fusion sequences have been associated with improved progression free survival in patients with castrate resistant prostate cancer treated with abiraterone treatment compared to ERG negative or ERG rearranged tumors [48]. In preclinical studies, SPINK1 expressing tumors have been shown to be susceptible to targeting by anti-SPINK1 antibodies, as well as inhibitions of the EGFR signaling pathway [49]. Therefore, there might be possible roles for assessment of ERG and SPINK1 expression in prostate cancer care in the future.

In summary, high expression of ERG and SPINK1 were associated with improved recurrence free survival in our multi-institutional cohort on univariate analysis. However, only SPINK1 over-expression remained significantly associated with improved RFS in multivariate models that took into account additional clinical and pathological parameters. Furthermore, neither biomarker was associated with differences in DSS or OS, although the number of events in the cohort was modest. When placed in context of other studies that relate expression of these biomarkers to clinical outcome, it is unlikely that either identifies molecular subtypes that are linked to prognosis. However, it is possible that when combined with other molecular biomarkers, ERG and SPINK1 could be useful in predicting outcome or predicting responses to therapy.

## Supporting Information

S1 File Raw clinical, pathological and staining data from the cohort. (XLSX)
